# High-Performance Planar Broadband Hot-Electron Photodetection through Platinum–Dielectric Triple Junctions

**DOI:** 10.3390/nano14191552

**Published:** 2024-09-25

**Authors:** Xiaoyan Yang, Yongmei Wang, Yaoyao Li, Weihao Cui, Junhui Hu, Qingjia Zhou, Weijia Shao

**Affiliations:** 1 School of Politics and Public Administration, Guangxi Normal University, Guilin 541004, China; xyyang@gxnu.edu.cn; 2 School of Physical Science and Technology & Guangxi Key Laboratory of Nuclear Physics and Technology, Guangxi Normal University, Guilin 541004, China; wangyongmei65@163.com (Y.W.); yaoyaoli@stu.gxnu.edu.cn (Y.L.); cuiwh@stu.gxnu.edu.cn (W.C.); hujh@mailbox.gxnu.edu.cn (J.H.); qingjiazhou@gxnu.edu.cn (Q.Z.)

**Keywords:** hot electrons, broadband absorption, detection, optical admittance

## Abstract

Recently, planar and broadband hot-electron photodetectors (HE PDs) were established but exhibited degraded performances due to the adoptions of the single-junction configurations and the utilizations of absorbable films with thicknesses larger than the electronic mean free path. In this work, we present a five-layer design for planar HE PDs assisted by triple junctions in which an ultrathin Pt layer dominates the broadband and displays strong optical absorption (>0.9 from 900 nm to 1700 nm). Optical studies reveal that the optical admittance matching between optical admittances of designed device and air at all interested wavelengths is responsible for broadband light-trapping that induces prominent energy depositions in Pt layers. Electrical investigations show that, benefitting from suppressed hot-electron transport losses and increased hot-electron harvesting junctions, the predicted responsivity of the designed HE PD is up to 8.51 mA/W at 900 nm. Moreover, the high average absorption (responsivity) of 0.96 (3.66 mA/W) is substantially sustained over a broad incidence angle regardless of the polarizations of incident light. The comparison studies between five-layer and three-layer devices emphasize the superiority of five-layer design in strong optical absorption in Pt layers and efficient hot-electron extraction.

## 1. Introduction

Extracting energetic electrons (i.e., hot electrons) generated in metals from the non-radiative decay of optical excitations has been shown to provide promising benefits in a variety of applications, including photocatalysis [1], optical sensing [2], photovoltaic [3], and photodetection [4]. In particular, hot-electron photodetection has been widely investigated due to the salient features of room-temperature operation, gap-free detection, and ultrafast response time [5,6,7,8,9,10]. Based on internal photoemission mechanism [11], hot-electron photodetectors (HE PDs) utilize metal-semiconductor Schottky junctions to extract photoexcited hot electrons and convert incidence energies into measurable photocurrent [12]. In general, broadband photoelectric responses are favorable for device operation. In the past decade, quite a few HE PDs with various metallic nanostructures have been experimentally demonstrated, exhibiting strong optical absorptions and efficient hot-electron extractions over a broad spectrum of wavelengths [13,14,15,16]. However, fabrications of complex nanostructures rely on expensive preparation technologies and nanostructured designs are confronted with difficulties in large-area application.

Unlike nanostructured HE PDs, purely planar counterparts have attracted increasing interest due to advantages of cost-effective fabrication and large-scale applications [17,18,19,20]. However, conventional planar HE PDs based on Au and Ag likely suffer from narrow-band photoelectric conversion because of discrete resonance wavelengths at which devices exhibit strong energy depositions in metals in which hot electrons arise [21,22,23,24,25,26]. Recently, absorbable materials with high refractive indexes, such as Ti [27] and TiN [28,29], have been integrated into planar Schottky junctions to realize strong optical absorptions over wide wavelength ranges, providing a feasible route toward broadband hot-electron photodetection. Unfortunately, the performances of these planar devices need to be further improved due to adoptions of single hot-electron harvesting junctions and contradiction between high absorption efficiencies and suppressed hot-electron transport losses. In detail, in these devices, thin absorbable films with thicknesses larger than electronic mean free paths are in contact with semiconductors to form single Schottky junctions, degrading hot-electron transport and collection efficiencies simultaneously. Therefore, suitable multiple-junction configurations in which ultrathin metal film dominates strong optical absorption over a wide wavelength range are highly desired for high-performance, planar, and broadband hot-electron photodetection.

In this work, the five-layer design of planar HE PDs has been proposed to realize broadband and efficient hot-electron extraction by triple Pt-TiO_2_ junctions, where an ultrathin Pt layer absorbs most of the energies of incident light over a wide wavelength range. Detailed optical studies revealed the inner relationships between the strong broadband absorptions (>0.9) and device admittances that are in close proximity to air admittance. Electrical investigations show that, benefiting from designed triple-junction architecture and broadband energy depositions in the ultrathin Pt layer, the average absorption and average responsivity of the designed device within the wavelength range of 900–1700 nm reach 0.96 and 3.66 mA/W, respectively. Furthermore, these outstanding optical and electrical responses are roughly maintained when incident angle increases from 0° to 50°. Our design is believed to meet the urgent need for high-performance, planar, broadband HE PDs and to promote the development of technology of hot-electron photoelectric conversion.

## 2. Results and Discussion

**Figure 1:**
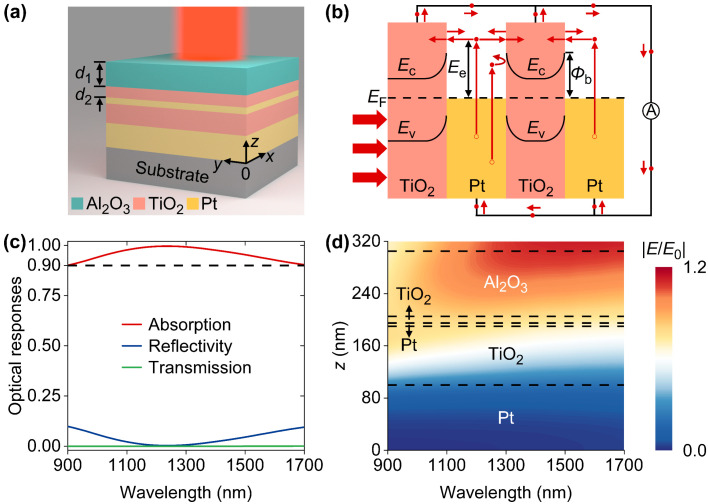
(**a**) Three-dimensional view of designed five-layer HE PD. (**b**) Energy diagram of triple Pt-TiO_2_ junctions for hot-electron extraction. (**c**) Spectral responses of the device. (**d**) Normalized electric field (|*E*/*E*_0_|) profiles as a function of wavelength.

As schematically shown in Figure 1a, the designed device is a multi-layer thin film stack that consists of an Al_2_O_3_ layer supported by two pairs of alternatively arranged Pt/TiO_2_ layers. The device is mounted on a silica substrate. The thicknesses of bottom Pt layer, top Pt layer, and TiO_2_ layer sandwiched by two Pt layers are 100 nm, 5 nm, and 90 nm, respectively. The thicknesses of Al_2_O_3_ layer and top TiO_2_ layer are denoted by *d*_1_ and *d*_2_, respectively. Figure 1b depicts the energy band diagram of the triple Pt-TiO_2_ junctions in which three consecutive electronic processes, namely, hot-electron generation, transport, and collection, are also shown. In specific, depositions of incidence energies into Pt layers induce the electronic transitions from occupied levels below the Fermi level (*E*_F_) to unoccupied higher levels whose energy exceeding *E*_F_ is denoted by *E*_e_. The generated hot electrons would diffuse toward Pt-TiO_2_ interfaces accompanied by transport losses due to electron–electron and electron–phonon scatterings. Considering that the electronic mean free path is about 11 nm in Pt [30], a fairly large number of hot electrons generated in top Pt layer can reach Pt-TiO_2_ layers without experiencing sufficient thermalization. Upon arriving at interfaces, hot electrons with energies larger than Schottky barriers (*Φ*_b_ = 0.73 eV) have sufficient energies to surmount barrier and thus have a chance to be injected into TiO_2_, while below-barrier (*E*_e_ < *Φ*_b_) ones would be blocked. The hot electrons entering successfully into TiO_2_ can be efficiently collected by the electrodes in contact with TiO_2_ layers and manifest as a photocurrent with the aid of external circuit. With the optical constants previously reported [31], we studied the spectral responses of the designed device with parameters of *d*_1_ = 100 nm and *d*_2_ = 10 nm in a finite-element platform [32], as shown in Figure 1c. It is found that the device exhibits broadband anti-reflection characteristics with a reflectivity of less than 0.1 at wavelengths ranging from 900 nm to 1700 nm. Given the negligible device transmission due to an optically thick bottom Pt layer, low broadband reflectivity leads to high absorption efficiencies (>0.9) from 900 nm to 1700 nm. In particular, an extremely low reflectance (~0) at 1233 nm corresponds to nearly perfect absorption. To investigate the broadband and strong absorption, the spatial distributions of electric fields (|*E*|) normalized by the electric fields (|*E*_0_|) of incident light were studied, as shown in Figure 1d. Obviously, the electric fields of incident light at each wavelength are predominantly confined to the Al_2_O_3_ layer, resulting in broadband anti-reflection.

**Figure 2:**
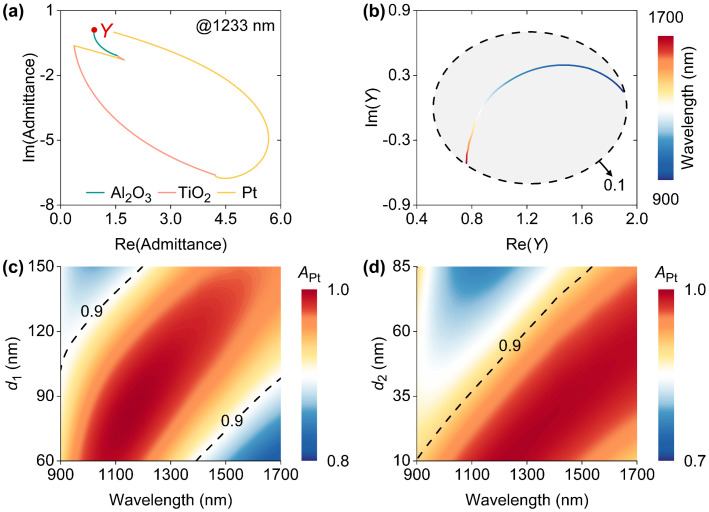
(**a**) Optical admittance locus of the designed HE PD at 1233 nm. Re(Admittance) and Im(Admittance) are the real and imaginary parts of admittance, respectively. (**b**) The wavelength-dependent device admittance (*Y*). The dashed ellipse in (**b**) represents iso-reflectance for 0.1 reflection. Contour maps of wavelength-dependent absorption (*A*_Pt_) in Pt layers as a function of *d*_1_ and *d*_2_. Three contour lines in (**c**,**d**) indicate absorption efficiencies of 0.9.

In order to gain more insights into obtained spectra, optical admittances that consist of real and imaginary parts have been calculated by the transfer matrix method [33]. As shown by the piecewise curves in Figure 2a, when the wavelength of incident light is 1289 nm, admittances vary rapidly as the thickness increases. In addition, the red dot in Figure 2a represents the admittance (*Y*) of the upper surface of the Al_2_O_3_ layer. *Y* can be identified as the device admittance. It is known that device reflectivity can be expressed by |(*Y* − 1)/(*Y* + 1)|^2^ [34]. Therefore, the extremely low reflectivity at 1233 nm can be attributed to the close proximity between *Y* and air admittance. Then, we investigated the correlations between *Y* and the wavelength of incident light, as shown by the colored curve in Figure 2b in which the dashed ellipse represents the iso-reflectance with a 0.1 reflection. It is evident that both parts of all *Y* fall within the gray area enclosed by the iso-reflectance curve, leading to the observed broadband light-trapping that induces strong absorption (*A*_Pt_) in absorbable Pt materials, particularly in the top Pt layer. We studied the dependences of *A*_Pt_ on two structural parameters, i.e., *d*_1_ and *d*_2_, as shown in Figure 2c,d, respectively. The dashed lines indicate the absorption efficiency of 0.9. It was found that with the increase in *d*_1_, the wavelength range for *A*_Pt_ > 0.9 increases first and then decreases. However, when *d*_2_ increases from 10 nm to 80 nm, the wavelength scope for *A*_Pt_ > 0.9 keeps decreasing. In other words, the structural parameters should be carefully chosen to ensure that *A*_Pt_ is always larger than 0.9 in the wavelength range from 900 nm to 1700 nm.

**Figure 3:**
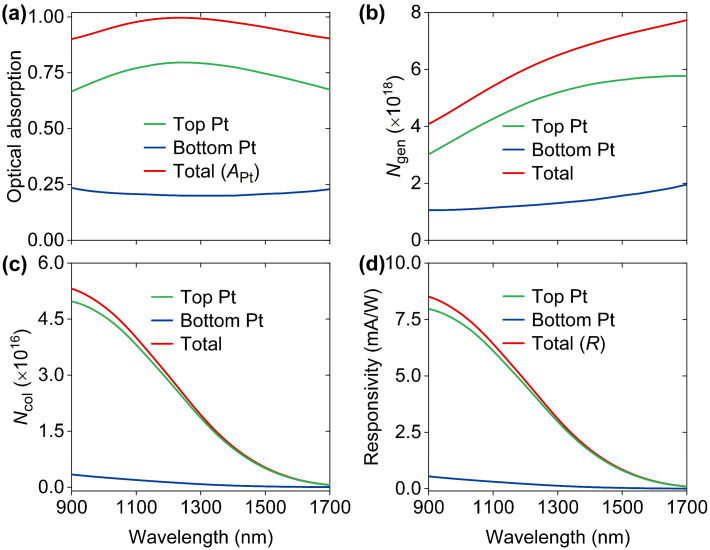
(**a**) Absorption contributions by two Pt layers and wavelength-dependent absorption (*A*_Pt_) in both two Pt layers. (**b**) Detailed wavelength-dependent flux (*N*_gen_) of generated hot electrons. (**c**) Spectra of flux (*N*_col_) of collected hot electrons. (**d**) Two responsivity components and total responsivity (*R*) as a function of wavelength.

After primary optical investigations, we turned our attention to the device electrical responses by a well-established photoelectric model in which the power (*P*_in_) of incident light at each wavelength is 1 W [35]. Firstly, the optical absorptions associated with Pt layers were studied, as shown in Figure 3a. It was found that *A*_Pt_ is always larger than 0.9 at each wavelength ranging from 900 nm to 1700 nm, indicating a good prospect in broadband photoelectric conversion. In addition, as mentioned above, although the top Pt layer is much thinner than the bottom Pt layer, absorption efficiencies in the top Pt layer are remarkably larger than those in the bottom layer. Considering that the generation rates are proportional to absorption efficiencies, the populations (*N*_gen_) per second in the top Pt layer are significantly larger than those in the bottom Pt layer, as shown in Figure 3b. Based on *N*_gen_, we obtained the wavelength-dependent flux (*N*_col_) of collected hot electrons by taking hot-electron transport loss within two Pt layers and hot-electron interfacial reflection at the Pt-Si interfaces into account, as shown in Figure 3c. It was found that the top Pt layer dominates the hot-electron collection. In addition, *N*_col_ reduces as wavelength increases due to the decrease in the proportions of above-barrier hot electrons, leading to a reduction in internal quantum efficiencies [36]. Eventually, we can calculate the wavelength-dependent responsivity (*R*) with *N*_col_ by

(1) $$R=\frac{eN_{\mathrm{col}}}{P_{\mathrm{in}}}$$

where *e* is the charge on the electron. As shown in Figure 3d, the hot electrons from the top Pt layer mainly contribute to the photocurrent due to the high absorption efficiency in the ultrathin layer that can significantly suppress hot-electron transport losses and enhance hot-electron collection efficiencies. Predicated *R* is up to 8.51 mA/W at 900 nm and decreases with the increase in wavelength. It is worth noting that electrical performances of the designed device are better than those of previously reported devices [14,15,16,17,18,19,20,21].

**Figure 4:**
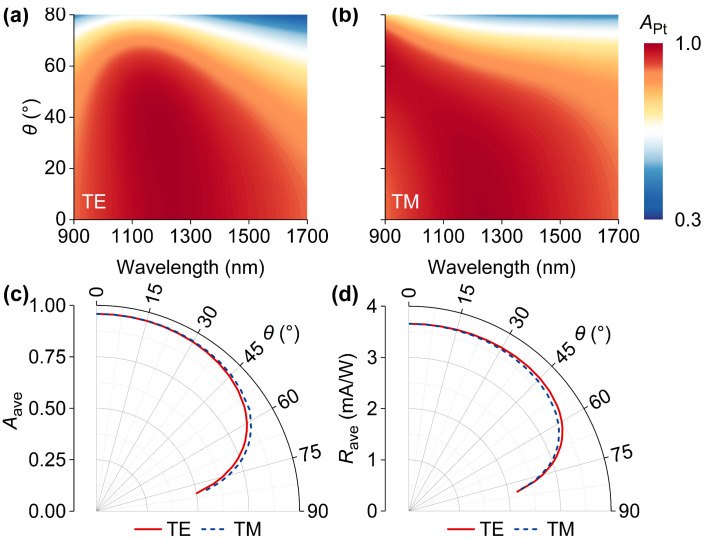
*A*_Pt_ as a function of incident angle (*θ*) under (**a**) transverse electric (TE) and (**b**) transverse magnetic (TM) incidences. Angular performances in (**c**) average absorption (*A*_ave_) in Pt layers and (**d**) average device responsivity (*R*_ave_) over the wavelength range from 900 nm to 1700 nm.

In order to comprehensively assess device performances, wavelength-dependent *A*_Pt_ in the cases of oblique illuminations were studied. As shown in Figure 4a,b, when the incidence angle (*θ*) increases from 0° to 50°, the broadband and strong *A*_Pt_ is essentially maintained for both transverse electric (TE) and transverse magnetic (TM) incidences, respectively. When *θ* further increases to 80°, *A*_Pt_ significantly degrades across the studied wavelength range. To quantitatively describe the dependences of broadband photoelectric responses on *θ*, average absorption (*A*_ave_) in Pt layers and average device responsivity (*R*_ave_) were defined. *A*_ave_ and *R*_ave_ can be expressed by

(2) $$A_{\mathrm{ave}}=\frac{\int_{\lambda_{1}}^{\lambda_{2}}A_{\mathrm{Pt}}d\lambda }{\lambda_{2}-\lambda_{1}}$$

(3) $$R_{\mathrm{ave}}=\frac{\int_{\lambda_{1}}^{\lambda_{2}}Rd\lambda }{\lambda_{2}-\lambda_{1}}$$

in which *λ*_2_ = 1700 nm and *λ*_1_ = 900 nm. When the device is illuminated by a normal incident light, the predicted *A*_ave_ and *R*_ave_ are 0.96 and 3.66 mA/W, respectively. As shown in Figure 4c,d, when *θ* is less than 50°, both *A*_ave_ and *R*_ave_ are almost irrelevant to *θ* under the illuminations of both TE- and TM-polarized light, respectively. Although *A*_ave_ and *R*_ave_ decreased as *θ* increased, the rough insensitivities of photoelectric performances to *θ* guarantee the flexibility of device operation. Moreover, in general, the angular behaviors of *A*_ave_ and *R*_ave_ under TE incidence are consistent with those under TM incidence, indicating that incidence polarization is a minor factor for planar and broadband hot-electron photodetection.

**Figure 5:**
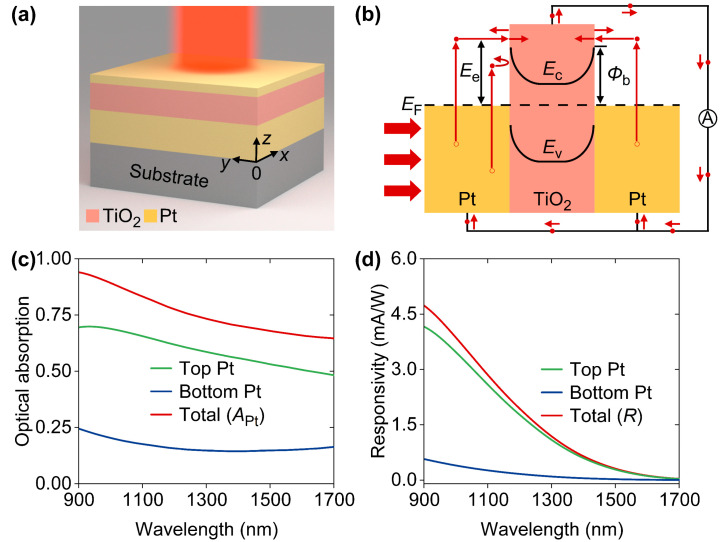
(**a**) Schematics of three-layer design of HE PDs for comparison studies. (**b**) Energy diagram of double Pt-TiO_2_ hot-electron harvesting junctions. (**c**) Absorption and (**d**) responsivity spectra relevant to reference device.

Finally, for the sake of comparison, we investigated the photoelectric responses of a reference device obtained by removing the Al_2_O_3_ and the top TiO_2_ layers of the designed device, as shown in Figure 5a. For simplicity, both devices share a set of symbols for physics quantities, such as *A*_Pt_, *R*, and *R*_ave_. As indicated in Figure 5b, the physics governing hot-electron extractions in both devices are identical, but the hot-electron harvesting junctions of reference device are less than those of the designed device. Moreover, the prominent difference of refractive indexes between air and the top Pt layer for the reference device may assist incident light in reflecting off the device. In consequence, as shown in Figure 5c, although there are considerable absorptions, *A*_Pt_ decreases when compared to the designed device, where the refractive index gently jumps from air to Pt by inserting two dielectric layers with moderate refractive indexes. It was also found that optical absorptions in the top Pt layer are also dominant but undergo non-ignorable reductions due to the reflection properties caused by the top Pt layer. Figure 5d shows that responsivity from the top Pt layer mainly contributes to total responsivity (*R*), which decreases to 4.73 mA/W at 900 nm. Moreover, the *R*_ave_ of reference device is only 1.66 mA/W, indicating the importance of strong absorption in the ultrathin Pt layer and multi-junction configuration associated with the designed device.

## 3. Conclusions

To summarize, Pt-involved triple junctions in a five-layer HE PD have been demonstrated to achieve strong optical absorption (>0.9) and efficient hot-electron extraction in a wide wavelength range from 900 nm to 1700 nm. The broadband and strong energy depositions in Pt layers have been elucidated by the prominent light-trapping induced by optical admittance matching between the designed device and air. Due to the triple-junction configuration and dominant absorption in an ultrathin Pt layer, the responsivity of the designed device is up to 8.51 mA/W at 900 nm, with an average responsivity that reaches as high as 3.66 mA/W. In addition, the designed device possesses good operation flexibility due to the insensitivity of photoelectric performances to the incident angle under both TE and TM incidences. The photoelectric performances of a three-layer device were studied to emphasize the advantages of boosted hot-electron transport and collection efficiencies originating from massive hot electrons generated in the ultrathin top Pt layer that is adjacent to two TiO_2_ layers forming two junctions. Based on the broadband absorption of designed thin film stacks, we forecast potential utilization in radiative coolers [37].

## Data Availability

The raw data supporting the conclusions of this article will be made available by the authors on request.
